# Targeted migration of bone marrow mesenchymal stem cells inhibits silica-induced pulmonary fibrosis in rats

**DOI:** 10.1186/s13287-018-1083-y

**Published:** 2018-12-04

**Authors:** Xiaoli Li, Guoliang An, Yan Wang, Di Liang, Zhonghui Zhu, Lin Tian

**Affiliations:** 1 Beijing Tropical Medicine Research Institute, Beijing Friendship Hospital, Capital Medical University, Beijing, China; 2 Department of Occupational and Environmental Health, School of Public Health, Capital Medical University, No. 10, Xi toutiao outside You anmen, Beijing, 100069 China

**Keywords:** Bone marrow mesenchymal stem cells, Pulmonary fibrosis, Silicosis, Migration

## Abstract

**Background:**

Silicosis is a common occupational disease, characterized by silicotic nodules and diffuse pulmonary fibrosis. We demonstrated an anti-fibrotic effect of bone marrow mesenchymal stem cells (BMSCs) in silica-induced lung fibrosis. In the present study, we sought to clarify the homing ability of BMSCs and the specific mechanisms for their effects.

**Methods and results:**

The biodistribution of BMSCs was identified by near-infrared fluorescence (NIRF) imaging in vivo and in vitro. The results showed that BMSCs labeled with NIR-DiR dyes targeted silica-injured lung tissue, wherein they reached a peak at 6 h post-injection and declined dramatically by day 3. Based on these findings, a second injection of BMSCs was administered 3 days after the first injection. The injected BMSCs migrated to the injured lungs, but did not undergo transformation into specific lung cell types. Interestingly, the injection of BMSC-conditioned medium (BMSCs-CM) significantly attenuated silica-induced pulmonary fibrosis. The collagen deposition and number of nodules were decreased in lung tissues of BMSCs-CM-treated rats. In parallel with these findings, the mRNA levels of collagen I, collagen III, and fibronectin, and the content of transforming growth factor (TGF)-β1 and hydroxyproline were decreased in the BMSCs-CM-treated group compared with the silica group. In addition, alveolar epithelial markers were upregulated by BMSCs-CM treatment.

**Conclusions:**

BMSCs migrated to injured areas of the lung after silica instillation and attenuated pulmonary fibrosis. The anti-fibrotic effects of BMSCs were mainly exerted in paracrine manner, rather than through their ability to undergo differentiation.

**Electronic supplementary material:**

The online version of this article (10.1186/s13287-018-1083-y) contains supplementary material, which is available to authorized users.

## Background

Silicosis is an important fibrotic pulmonary disease induced by inhalation of crystalline silica dust. It is an occupational disease worldwide, and no cure has been established to date [1]. Treatment options currently focus on alleviating symptoms, limiting inflammation, and preventing complications. Clearly, it is desirable to develop novel and promising therapeutics to halt the fibrogenic process.

Bone marrow mesenchymal stem cells (BMSCs) are considered excellent candidates for stem cell-based therapy, based on their immense plasticity, paracrine mechanism of action, and immunomodulatory properties for treatment of previously incurable disorders [2–4]. BMSCs also have a homing ability that may contribute to their therapeutic effects, because they can preferentially migrate to injury sites and exert functional effects for organ injury. Kidder D [5] investigated BMSCs accumulation in the murine spleen and found that BMSCs interacted with splenocytes to ameliorate renal injury. Liu et al. [6] indicated that CdSe/ZnS quantum dot-labeled MSCs were homed to the pathological pancreas initiatively in diabetic rats. Several studies suggested that BMSCs can actively home to micro metastatic regions to halt carcinogenic progression [7]. Collectively, these properties pave the way for application of BMSCs to the treatment of fibrotic pulmonary diseases. Multiple studies have recorded therapeutic effects of BMSCs on pulmonary fibrosis, including reduction in lung collagen deposition, downregulation of proinflammatory and angiogenic cytokines, and decrease in oxidative stress [8–11].

We previously demonstrated that BMSCs inhibited pulmonary fibrosis and protected damaged epithelial cells in a rat model of silica-induced pulmonary fibrosis [12]. Therefore, it was considered that BMSCs were able to engraft with high efficacy in the lung, make direct contact with the injured lung epithelium, and repair or restore the damaged tissue. However, BMSCs did not give rise to alveolar epithelial cells directly when they were co-cultured with rat alveolar epithelial type II (ATII) cells in vitro following silica-induced injury. Thus, it remains to be established whether similar findings are detected in silica-injured rat lungs and whether BMSCs are actively recruited to the injured lung. The exact protective mechanism also requires further exploration.

Near-infrared fluorescence (NIRF) imaging is a non-invasive technique that can specifically visualize molecular and cellular processes in living animals. Because of its non-radioactive nature and high sensitivity, the technique has been widely employed. To establish an optimized drug-delivery system for central nervous system diseases, NIRF imaging was used to identify the biodistribution of liposomes in the mouse brain [13]. Furthermore, the distribution of DiR-labeled murine embryonic stem cells was monitored in gastric tumor-bearing mice by NIRF imaging [14]. Using this technique, Leng et al. [15] showed that MSCs can inhibit breast cancer progression by suppressing angiogenesis.

To determine whether BMSCs were recruited to the site of silica-induced injury in vivo, the behavior of transplanted BMSCs was traced in the rat lung by NIRF imaging in the present study. We further focused on the underlying mechanism, differentiation or paracrine effects, for BMSC involvement in amelioration of silica-induced pulmonary fibrosis. To investigate the potential beneficial paracrine effect, conditioned medium from BMSCs (BMSCs-CM) was injected into silica-induced fibrosis model rats. Finally, the results demonstrated that BMSCs migrated from the circulation into the damaged lung and inhibited silica-induced pulmonary fibrosis via paracrine soluble factors.

## Methods

### Silica

The crystalline silica (size distribution 95% < 5 μm, purity > 99%) used in this study was purchased from Sigma-Aldrich (St Louis, MO, USA). The silica suspensions (50 mg/ml) were autoclaved (0.1 Mpa, 120 °C, 20 min). Added with penicillin (5000 U/ml), they were sonicated for 10 min before use.

### Animals and ethics statement

Specific pathogen-free Wistar rats (male 180~200 g, female 200~240 g) were purchased from Vital River Laboratory Animal Technology Co. Ltd. (Beijing, China). Rats were maintained at a temperature-controlled room (24 ± 1 °C) with a 12:12-h light/dark cycle and received free access to food and water. Before starting the study, they were adapted to the conditions for 7 days. All experimental procedures were approved by the Laboratory Animal Care and Use Committee at Capital Medical University (Approval number AEEI-2015-033) and met requirements described in the National Institute of Health Guide for the Care and Use of Laboratory Animals.

### Preparations of primary male rat BMSCs cultures

Primary BMSCs were isolated following the procedure previously described [16]. In brief, the bone marrow, collected from the femur and tibiae of male Wistar rats, was incubated in alpha-modified minimum essential medium (a-MEM; Hyclone, Logan, UT, USA) containing 14% fetal bovine serum (Gemini Bio-Products, West Sacramento, CA, USA), 100 IU/ml penicillin, and 100 mg/ml streptomycin (KeyGEN, Nanjing, China) at 37 °C in a humidified atmosphere containing 5% CO_2_. Passage 3 BMSCs were used in the present experiment. The isolated cells were characterized by flow cytometric analysis of specific panel of markers, including CD90 (Becton Dickinson, Mississauga, ON, Canada), CD11b, CD44, and CD45 (eBioscience, San Diego, CA, USA). To analyze the potential of differentiation, BMSCs were induced in adipogenesis or osteogenesis differentiation medium for 21 days in vitro (Additional file 1).

### Labeling BMSCs with DiR

BMSCs were labeled with a lipophilic, near-infrared fluorescent dye 1,1'-dioctadecyltetramethyl indotricarbocyanine iodide (DiR; Life Technologies, Carlsbad, CA, USA). Primary BMSCs were suspended at a concentration of 2 × 10^6^ cells/ml and incubated with DiR buffer for 1 h at 37 °C according to the manufacturer’s protocol. Thereafter, BMSCs were washed twice with phosphate-buffered saline (PBS) before infusion for unlabeled cells. The labeled cells (2 × 10^6^ in 1 ml saline) were injected by tail vein injection.

### Cell viability of BMSCs

Cellular viability of BMSCs was assessed by 3-(4, 5-dimethylthiazol-2-yl)-2, 5-diphenyltetrazoliumbromide (MTT) assays as previously described [12]. BMSCs (5 × 10^3^ cells/well) were seeded in 96-well plates, cultured for 24 h, then added with 0, 2.5, 5, or 10 μg/ml DiR buffer for 24 h, 48 h, and 72 h, respectively. MTT reagent (20 μl, 5 mg/ml, Sigma-Aldrich) was treated with each well. After incubated at 37 °C for 4 h, a dimethyl sulfoxide (DMSO) solution was added into each well by gentle shaking. The absorbance was measured at 550-nm wavelengths.

### In vivo imaging of transplanted BMSCs

In order to assess the biodistribution of BMSCs in silica models, female adult rats were randomly divided into three groups (*n* = 18 per group): (1) the control group received saline, (2) the DiR-BMSCs + control group received saline and DiR-labeled BMSCs, and (3) the DiR-BMSCs + silica group received silica and DiR-labeled BMSCs. Rats were intratracheally injected with silica suspension (1 ml of 50 mg/ml/rat) to produce silica-induced pulmonary fibrosis, as previously described by Yang et al. [17]. Rats were anesthetized with 2.5% isoflurane and were monitored using the in vivo imaging systems at 1 h, 6 h, 24 h, 3 days, 15 days, and 30 days after tail intravenous injection of DiR-labeled BMSCs (2 × 10^6^ cells in 1 ml saline). The serial fluorescence images were also obtained in ex vivo organs (lung, live, kidney, spleen, heart) at designated time points. In order to reduced autofluorescence from animals at higher wavelengths, the ideal filter conditions for DiR imaging were adopted an excitation/emission spectrum in the near infrared (750/780 nm) [18].

### Animal experiments to investigate anti-fibrotic efficacy

To assess the effect of BMSCs on inhibiting the pulmonary fibrosis processes, female Wistar rats were divided into the control, silica, and BMSCs groups (*n* = 8 per group). According to the dose and timing of BMSCs administration, BMSCs were divided into four groups: BMSCs-1, BMSCs-2, BMSCs-3, and BMSCs-4 group*.* BMSCs in 1 ml saline were injected via the tail vein: BMSCs-1 (1 × 10^6^ cells, day 1), BMSCs-2 (1 × 10^6^ cells, days 1 and 4), BMSCs-3 (2 × 10^6^ cells, day 1), or BMSCs-4 (2 × 10^6^ cells, days 1 and 4). Rats in the control and silica groups were injected with saline (1 ml/rat) to match the schedule. The rats were sacrificed on day 15 after silica instillation. Lung or body weight was measured separately, and the lung/body weight ratio that represented the toxic effect of silica was calculated [19].

### BMSCs-CM generation

BMSCs (2 × 10^6^) were cultured in 10-cm diameter culture dishes, then washed three times with PBS, following which they were incubated in 10 ml α-MEM for 24 h. BMSCs-CM was collected and then centrifuged at 1500*g* for 10 min to remove cell debris. BMSCs-CM was further concentrated using Amicon® Ultra-15 centrifugal filter devices through 3-kDa molecular weight cutoff (Millipore, Billerica, MA) following the manufacturer’s instructions.

### The anti-fibrotic role of BMSCs-CM in rats

Female Wistar rats were divided into four groups (*n* = 8): the control group, silica group, silica + BMSCs group, and silica + BMSCs-CM group. On days 1 and 4 following the intratracheal administration of silica, they were injected intravenously 1 ml saline, 1 ml saline, 1 ml BMSCs (2 × 10^6^/ml), or 1 ml BMSCs-CM, respectively. Animals were sacrificed on day 15 or day 30, and the lung tissues were collected for further analysis.

### HE and Masson staining

The lungs were used for pathological examination. They were fixed by immersion in 10% neutral buffered formalin, embedded in paraffin, and approximately cut into 5-μm-thick sections. The sections were stained with H&E or Masson’s trichrome to evaluate histopathological detection. Lung morphologic changes were visualized with a light microscope (Olympus D72, Japan) at × 100 and × 200 magnifications. In addition, histological scoring was assessed by the extent of fibrosis in each field as previously described [16]. Semi-quantitative histopathologic determination of fibrosis was evaluated by Image-Pro Plus 6.0 software (Media Cybernetics, Rockville, MD, USA) according to the percentage of collagen (the blue area) [20].

### Determination of TGF-β1 levels in bronchoalveolar lavage fluid (BALF)

BALF was collected from these rats on days 15 and 30 after silica instillation as previously described [16]. Each BALF sample was centrifuged to remove the cells and debris. The supernatants was analyzed for the level of TGF-β1 with a rat TGF-β1 ELISA kit (Xitang, Shanghai, China).

### Assay of hydroxyproline

Specifically, the determination of lung fibrosis was evaluated through hydroxyproline (HYP) assays (HYP kit from Nanjing Jiancheng Biotechnology Co., Ltd., Nanjing, China). Experimental procedure was performed in compliance with the manufacturer’s protocol. The absorbance of sample was determined at 550 nm on a spectrophotometer. Data were quantified as milligrams of HYP per gram of wet lung weight.

### RNA isolation and quantitative real-time PCR (qPCR)

Total RNA of lung tissues was extracted with the SV total RNA isolation system (Promega Corporation, Madison, WI, USA) according to the manufacturer’s protocol. Reverse transcription was used Transcript First-strand cDNA Synthesis SuperMix (TransGen Biotech, Beijing, China). The DNA was extracted from lung with Solarbio DNA extraction kit (Beijing Solarbio Science & Technology Co. Ltd., Beijing, China). DNA or cDNA was synthesized using with a SYBR Green RT-PCR Kit (TransGen Biotech) for collagen I, collagen III, FN, Sry, AQP-5, and SP-C. The sequences of specific primers were presented in Table 1. The cycling conditions for qPCR were 94 °C for 30 s, followed by 40 cycles of 94 °C for 5 s, 60 °C for 15 s, and 72 °C for 10 s.

**Table 1:** Primer sequences for qPCR

Gene name	Forward (5′-3′)	Reverse (5′-3′)
Collagen I	CAATGGCACGGCTGTGTGCG	CACTCGCCCTCCCGTCTTTGG
Collagen III	TGAATGGTGGTTTTCAGTTCAG	GATCCCATCAGCTTCAGAGACT
FN	TGACAACTGCCGTAGACCTGG	TACTGGTTGTAGGTGTGGCCG
AQP-5	AAGGAGGTGTGCTCCCTTGCC	TCAGTGTGCCGTCAGCTCGAT
SP-C	GTCCTTGTCGTCGTGGTGAT	AGGTAGCGATGGTGTCTGTGT
Sry	CATCGAAGGGTTAAAGTGCCA	ATAGTGTGTAGGTTGTTGTCC
β-Actin	GTCAGGTCATCACTATCGGCAAT	AGAGGTCTTTACGGATGTCAACGT

*qPCR* quantitative real-time PCR, *FN* fibronectin, *Sry* sex-determining region Y, *AQP-5* aquaporin-5, *SP-C* surfactant protein-C

### Immunofluorescence

The lung tissues were extracted and immersed with 4% paraformaldehyde in PBS overnight, then cut into 20-μm-thick sections with a microtome. After blocked with 5% donkey serum in PBS for 30 min, sections were incubated at 4 °C overnight with primary antibodies as follows: goat anti-AQP-5 (quaporin-5, 1:50, Santa Cruz biotechnology, USA) and rabbit anti-RBMY (RNA-binding gene on Y chromosome, 1:50, Santa Cruz biotechnology, USA). After that, the sections were incubated with secondary antibody: donkey anti-goat IgG (Alexa Fluor® 647, Abcam, Cambridge, MA, USA) and donkey anti-rabbit IgG (Alexa Fluor® 647 Conjugate, Cell Signaling Technology Inc., Beverly, MA, USA) for 1 h at room temperature. Cell nuclei were stained with 4′-6-diamidino-2-phenylindole (DAPI). Images were obtained using Confocal Laser Scanning Microscopy (Nikon, Tokyo, Japan). The fluorescence intensity in each image was quantified by Image-Pro Plus 6.0 software.

### Statistical analysis

All results were acquired at least three times. Experimental data were presented as the mean ± standard deviation. For multiple group comparisons, one-way analysis of variance was performed, followed by the Student-Newman-Keuls post hoc test. All statistical analyses were carried out using SPSS 18.0 software (SPSS Inc., Chicago, IL, USA). A *p* value of less than 0.05 was set as statistically significant.

## Results

### Cytotoxicity of DiR in vitro

**Fig. 1:**
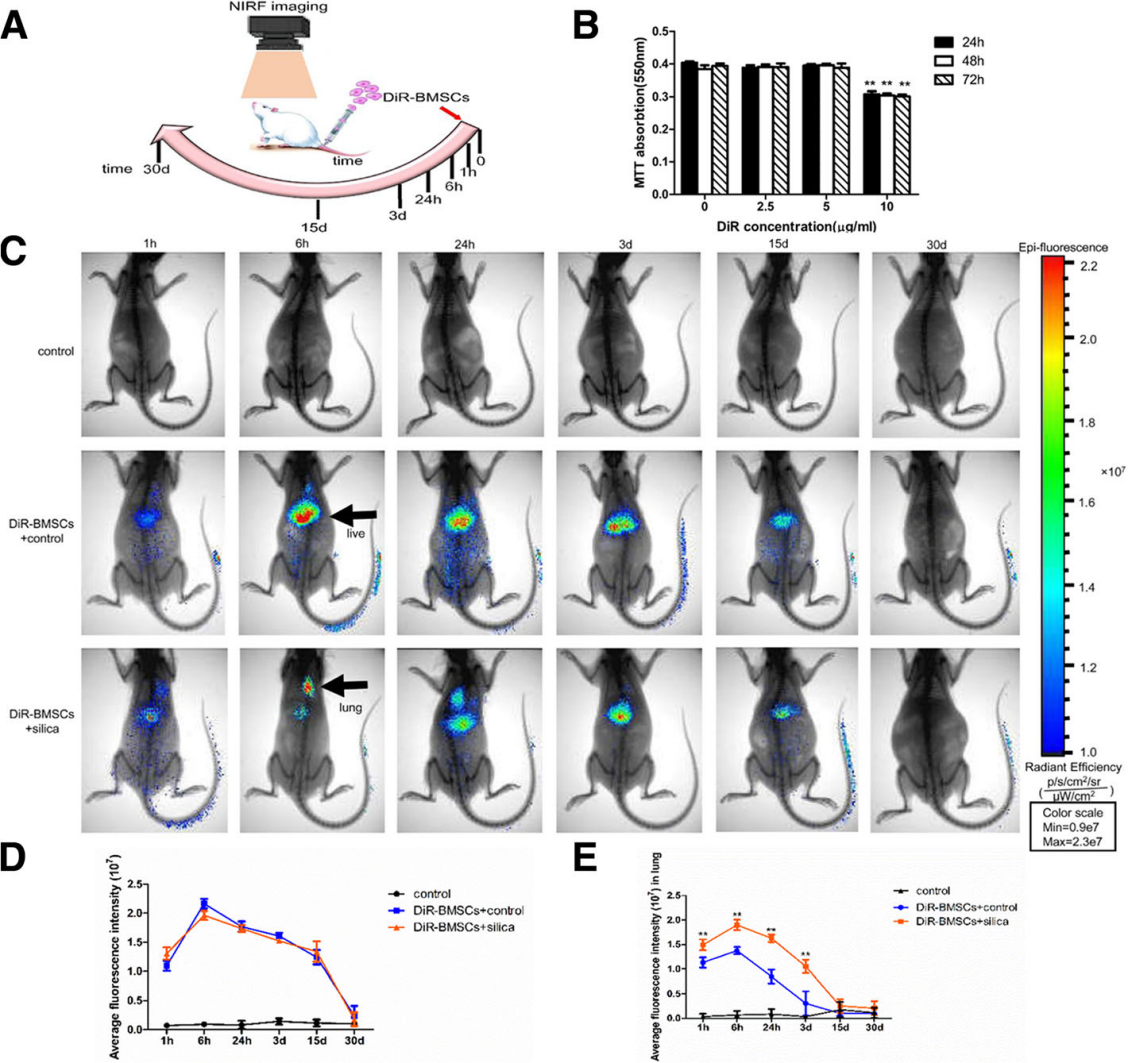
Homing of BMSCs to the silica-injured lung. **a** A diagram of experimental protocol. Rats were monitored using an in vivo imaging system at 1 h, 6 h, 24 h, 3 days, 15 days, and 30 days after transplantation of DiR-BMSCs. **b** Cytotoxicity of DiR in vitro. Relative cell viabilities of BMSCs were measured after being incubated with various doses DiR for 24 h, 48 h, and 72 h. Up to 5 μg/ml, DiR did not change cell viability. **p < 0.01 compared with 0 μg/ml group. **c** Whole-body imaging was monitored with the in vivo imaging system. DiR fluorescence intensity reached a peak at 6 h, declined dramatically by day 3, reduced over a long period (at least 15 days), and disappeared by day 30. BMSCs were predominantly distributed to the liver region in the DiR-BMSCs + control group, while many cells were accumulated in the thoracic (lung) region in the DiR-BMSCs + silica group. Scale bar = 5 cm. **d**, **e** Quantification of the fluorescence intensity in the whole body and in the lung. Much more cells were accumulated in the thoracic (lung) region in the DiR-BMSCs + silica group compared with the DiR-BMSCs + control group. The data were presented as the means ± SD. **p < 0.01 versus the DiR-BMSCs + control group

To select the optimal DiR concentration for label BMSCs, BMSCs were exposed to various concentrations of DiR (0~10 μg/ml). MTT assays showed that concentrations below 5 μg/ml did not alter the cell viability compared with control cells, while 10 μg/ml DiR caused a significant decrease in cell viability at (*p* < 0.01 for all each time points; Fig. 1b). Thus, 5 μg/ml appeared to be the optimal concentration for DiR, because it was nontoxic toward BMSCs and produced strong fluorescence signals for imaging and tracking of cells in vivo.

### Distribution of BMSCs in vivo (ability to home to the silica-injured lung)

To track the distribution of DiR-BMSCs in a rat model of silicosis fibrosis, whole-animal imaging was monitored with an in vivo imaging systems at designated time points (1 h, 6 h, 24 h, 3 days, 15 days, 30 days). As shown in Fig. 1c, the fluorescence intensity of DiR reached a peak at 6 h after transplantation of DiR-BMSCs and declined dramatically by day 3. Thereafter, the intensity gradually decreased over a relatively long period (at least 15 days) and ultimately disappeared by day 30. A quantitative evaluation of the fluorescence intensity is presented in Fig. 1d. For whole-body imaging, the fluorescence intensity in the DiR-BMSCs + control group was essentially the same as that in the DiR-BMSCs + silica group. However, the distribution of DiR-BMSCs in organs differed markedly between the two groups. BMSCs were predominantly distributed to the liver region in the DiR-BMSCs + control group, while many cells were accumulated in the thoracic (lung) region in the DiR-BMSCs + silica group. Compared with the DiR-BMSCs + control group, stronger fluorescence was detected in the lung in the DiR-BMSCs + silica group. To confirm the distribution of DiR-BMSCs, we quantified the fluorescence intensity for the DiR signal-positive area in the thoracic region. The results showed that the fluorescence intensity was significantly increased in the DiR-BMSCs + silica group compared with the DiR-BMSCs + control group at 1 h, 6 h, 24 h, and 3 days, respectively (*p* < 0.01; Fig. 1e). No DiR fluorescence signals were observed in the control group.

**Fig. 2:**
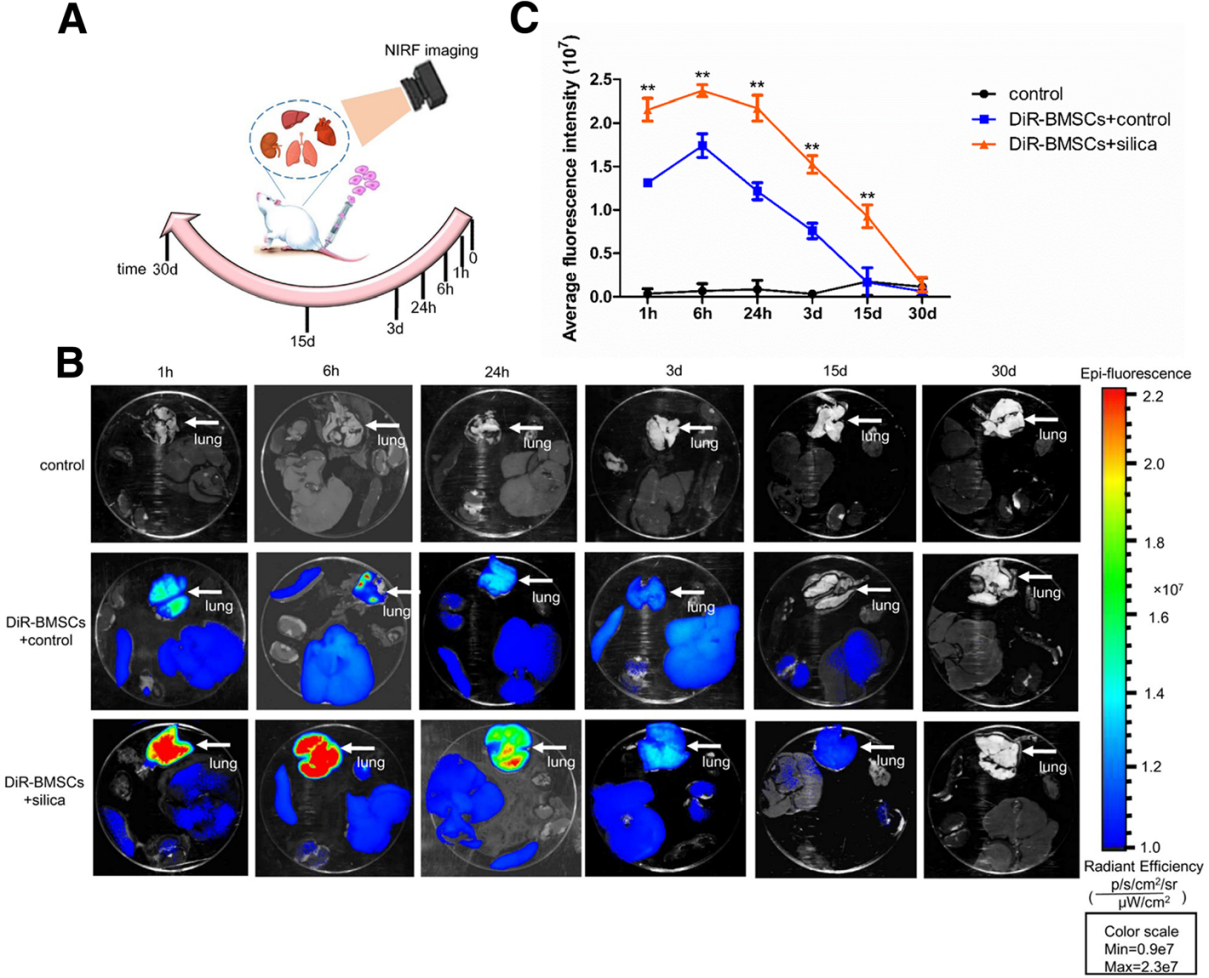
Ex vivo NIRF imaging of BMSCs in the lung. **a** A diagram of experimental protocol. The serial fluorescence images were also obtained in ex vivo organs (lung, live, kidney, spleen, heart) at designated time points. **b** DiR-BMSCs were predominantly distributed to the lung region, declined dramatically by days 3. Scale bar = 2 cm. **c** Quantification of the fluorescence intensity in the lung at 1 h, 6 h, 24 h, 3 days, 15 days, and 30 days. Compared with the DiR-BMSCs + control group, the fluorescence intensity in the lung was apparently increased in the DiR-BMSCs + silica group. The data were presented as the means ± SD. **p < 0.01 versus the DiR-BMSCs + control group

Ex vivo NIRF imaging was performed on internal organs collected from the rats at each time point. For both groups, the fluorescence intensity of DiR in the lung reached the maximum value at 6 h after injection and declined sharply by 3 days. At 2 weeks, very weak DiR signals were observed (Fig. 2b). Compared with the DiR-BMSCs + control group, the fluorescence intensity in the lung was apparently increased in the DiR-BMSCs + silica group. These observations, confirmed by quantification of the DiR signal intensity, were consistent with the whole-body imaging data described above (*p* < 0.01, Fig. 2c). Notably, DiR-BMSCs may tend to migrate to lung tissue injured by silica.

### Optimal dose and timing of BMSCs administration to attenuate silica-induced pulmonary fibrosis

To select the optimal delivery dose and timing, different numbers of BMSCs were transplanted into rats instilled with silica at different time points. To avoid pulmonary embolism induced by administration of high-dose BMSCs, a double low dose was used. NIRF imaging showed that the number of BMSCs was dramatically decreased in the lung at day 3. Therefore, a second dose of BMSCs was administered 4 days after silica instillation. As anticipated, the rats in BMSCs-4 group (2 × 10^6^ cells, days 1 and 4) exhibited significant reductions in inflammation and fibrosis markers in histological and biomolecular analyses.

**Fig. 3:**
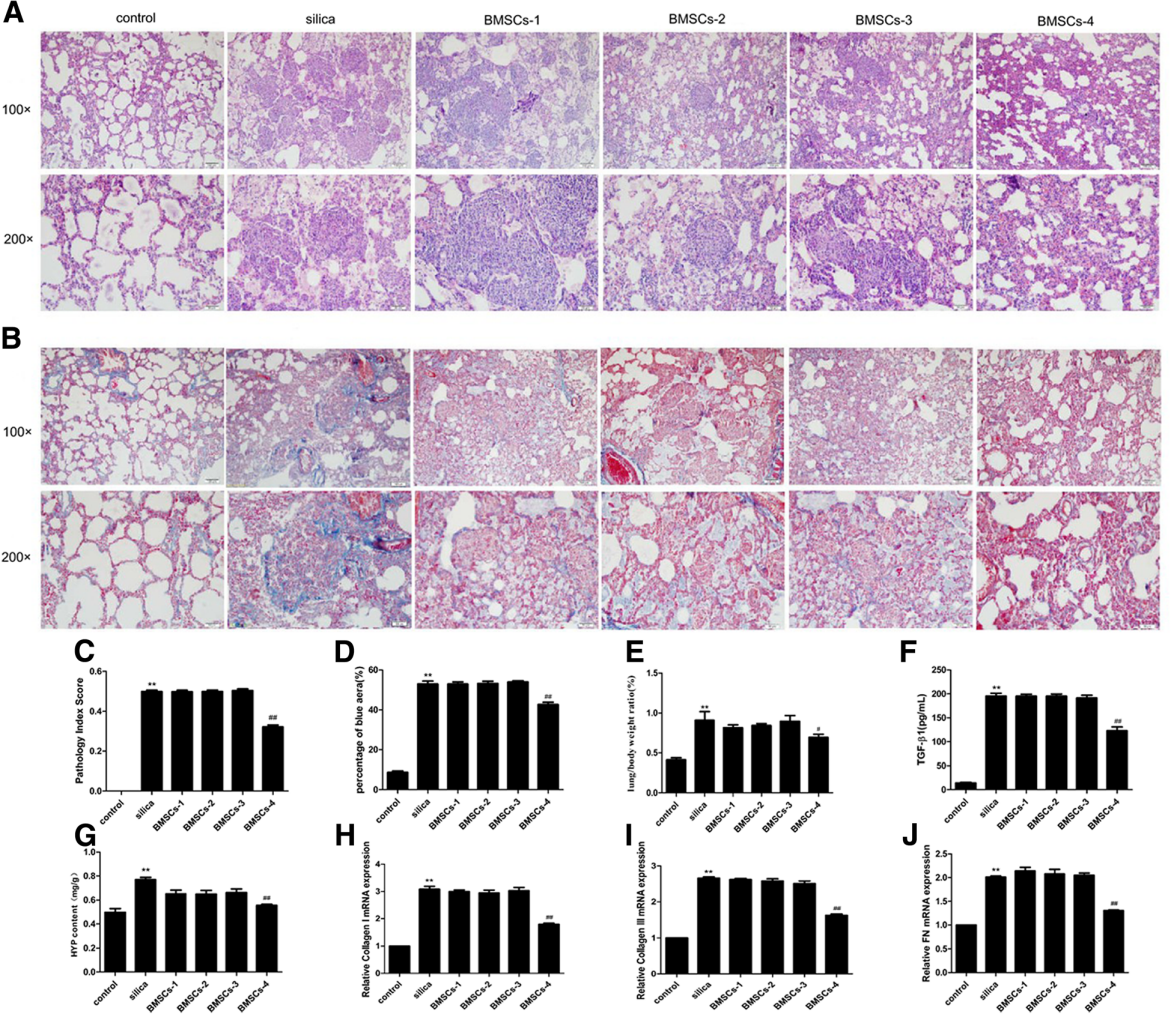
Optimal dose and timing of BMSCs administration to attenuate silica-induced pulmonary fibrosis**.** H&E staining (**a**) and Masson’s trichrome staining (**b**) in the rat lungs at 15 days after silica instillation. Light micrograph magnification × 100 and × 200. Scale bar, 100 μm and 50 μm. Quantitatively analyzed image stained in H&E (**c**) or Masson’s trichrome (**d**). The pathology index of lung tissue and the fibrotic areas were significantly increased in the silica group compared with the control group, but decreased in the BMSCs-4 group. The ratio of lung/body weight (**e**), level of TGF-β1 (**f**), content of HYP (**g**), expression of collagen I (**h**), collagen III (**i**), and FN (**j**) were significantly increased in the silica group in comparison with the control group, but were decreased in the BMSCs-4 group. BMSCs (2 × 10^6^ cells, days 1 and 4) attenuated silica-induced pulmonary fibrosis. Values are expressed as mean ± SD, *n* = 8. **p < 0.01 compared with the control group; ^#^*p* < 0.05, ^##^*p* < 0.01 compared with the silica group

In the histological evaluation, alveolar septal thickening, inflammatory cell infiltration, and cellular nodule formation were observed in the silica group, but these effects were decreased by treatment with BMSCs (2 × 10^6^ cells, days 1 and 4) (Fig. 3a, c). Masson staining, in which fibrotic collagen were stained blue, revealed that collagen deposition was increased in the silica group compared with the control group. In the BMSCs-4 group, the blue-stained areas were obviously decreased (Fig. 3b, d).

In parallel with the changes described above, the BMSCs in the BMSCs-4 group, but not the other three BMSCs groups, inhibited the silica-induced increases in fibrosis indicators, such as the ratio of lung/body weight, content of TGF-β1 and HYP, and expression of collagen I, collagen III, and fibronectin (FN) (*p* < 0.01; Fig. 3e–j). These findings verified that BMSCs attenuated silica-induced pulmonary fibrosis and that the optimal dose and timing were 2 × 10^6^ cells at days 1 and 4 after silica instillation. Therefore, this scheme was chosen for further experiments.

### BMSCs barely adopt lung epithelial cell phenotypes in vivo

**Fig. 4:**
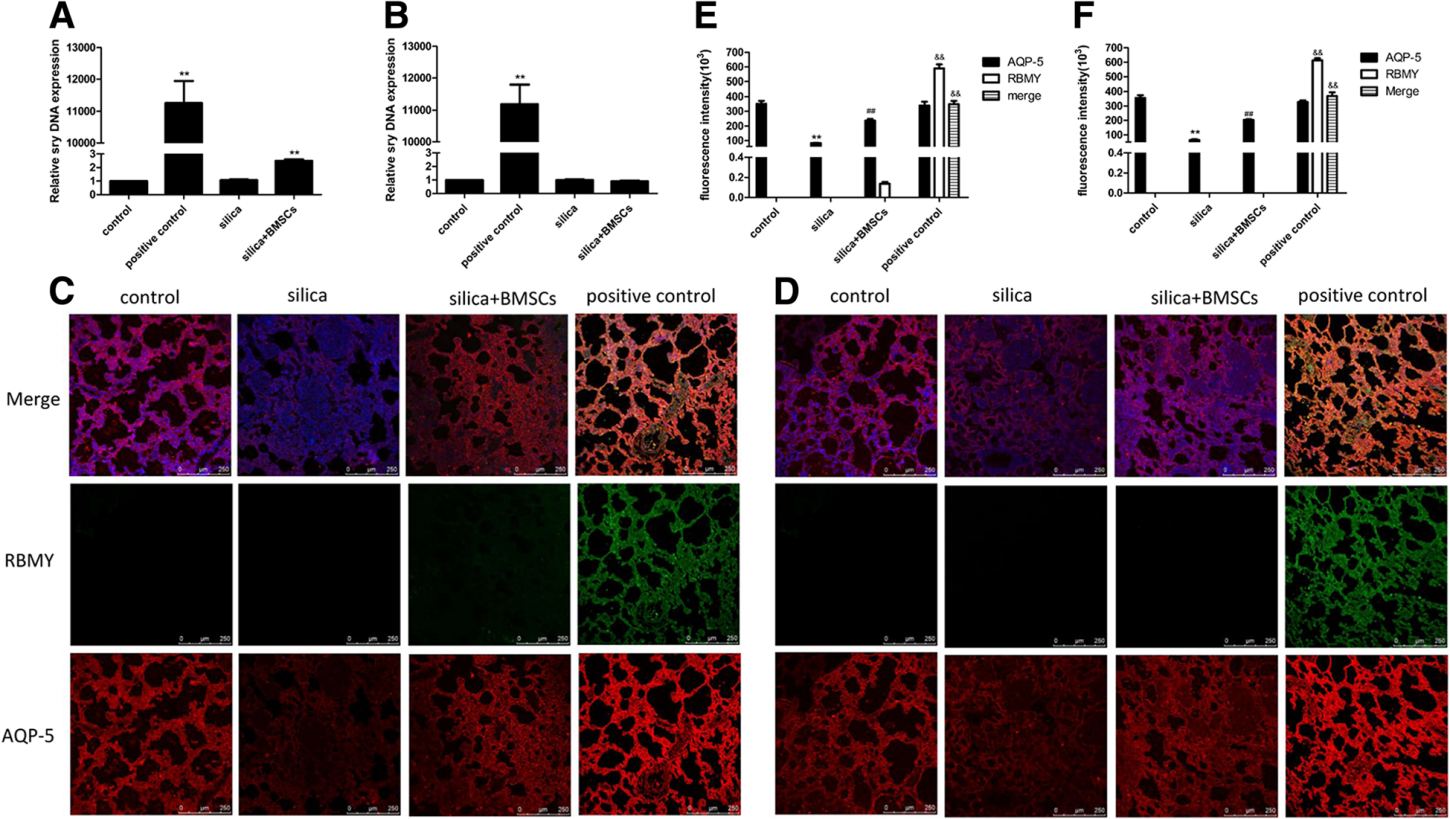
BMSCs barely adopt alveolar cell phenotypes. **a**, **b** Quantified engraftment levels of BMSCs in the lungs by real-time PCR analysis. Sry, representing male BMSCs DNA, was only shown up in the silica + BMSCs group on day 15 (**a**), but did not show up on day 30 (**b**). The male rat lung served as the positive control. The data were presented as the means ± SD. **p < 0.01 versus the control group. **c**, **d** Immunofluorescence (IF) of the lung sections was tested on day 15 (**c**) and on day 30 (**d**) by using a confocal microscopy. Nuclear staining (DAPI, blue), BMSCs (RBMY, green), and the ATI cells (AQP-5, red). The level of AQP-5 (red) protein expression was reduced in the silica group, but increased after treatment with BMSCs. Little RBMY protein (green) was detected in the lung tissue from the silica + BMSCs group on day15, but not on day 30. Colocalization, indicated by a yellow color, was not observed in the merged panel in the silica + BMSCs group. Male rat lung served as the positive control. Scale bar = 250 μm. **e**, **f** Quantification of fluorescence intensity was analyzed in each group on days 15 (**e**) and 30 (**f**). Values are expressed as mean ± SD, *n* = 8. **p < 0.01 compared with the control group; ^##^*p* < 0.01 compared with the silica group; ^&&^*p* < 0.01 compared with the silica + BMSCs group

As shown in Fig. 4a and b, the sex-determining region Y (Sry) gene (representing male BMSCs DNA) was not detected in the lungs of rats in the control and silica groups on days 15 and 30. A few BMSCs were detected in the lungs of the silica + BMSCs group on day 15, but no BMSCs were detected on day 30. These results show that BMSCs cannot achieve long-term lodgment in the injured area.

In our previous study, BMSCs did not differentiate into epithelial cells when co-cultured with silica-damaged rat ATII cells [12]. To determine whether BMSCs adopted lung cell phenotypes in silica-induced pulmonary fibrosis, BMSCs from male rats were intravenously injected into female rats. The lung tissue of the female rats was examined by immunofluorescence staining with antibodies against aquaporin (AQP)-5 (red fluorescence) for ATI cells and RBMY (on the Y chromosome; green fluorescence) for male BMSCs. Compared with the findings in the control group, the intensity of red fluorescence (AQP-5) was obviously decreased in the silica group, suggesting that ATI cells were damaged by silica. In contrast, the red fluorescence intensity was significantly increased after treatment with BMSCs, suggesting that BMSCs protected epithelial cells against silica-induced damage. For RBMY, the weak green fluorescence staining was observed in the lung tissue from the silica + BMSCs group on day15, but not on day 30. Colocalization, indicated by a yellow color, was not observed in the merged panel in the silica + BMSCs group (*p* < 0.01; Fig. 4c–f). These findings indicated that none of the epithelial cells was derived from BMSCs.

The above data suggested that the therapeutic effect of BMSCs may not be related to long-term survival and that BMSCs hardly differentiated into epithelial cells to repair damaged lung tissues.

### BMSCs-CM attenuated silica-induced pulmonary fibrosis

**Fig. 5:**
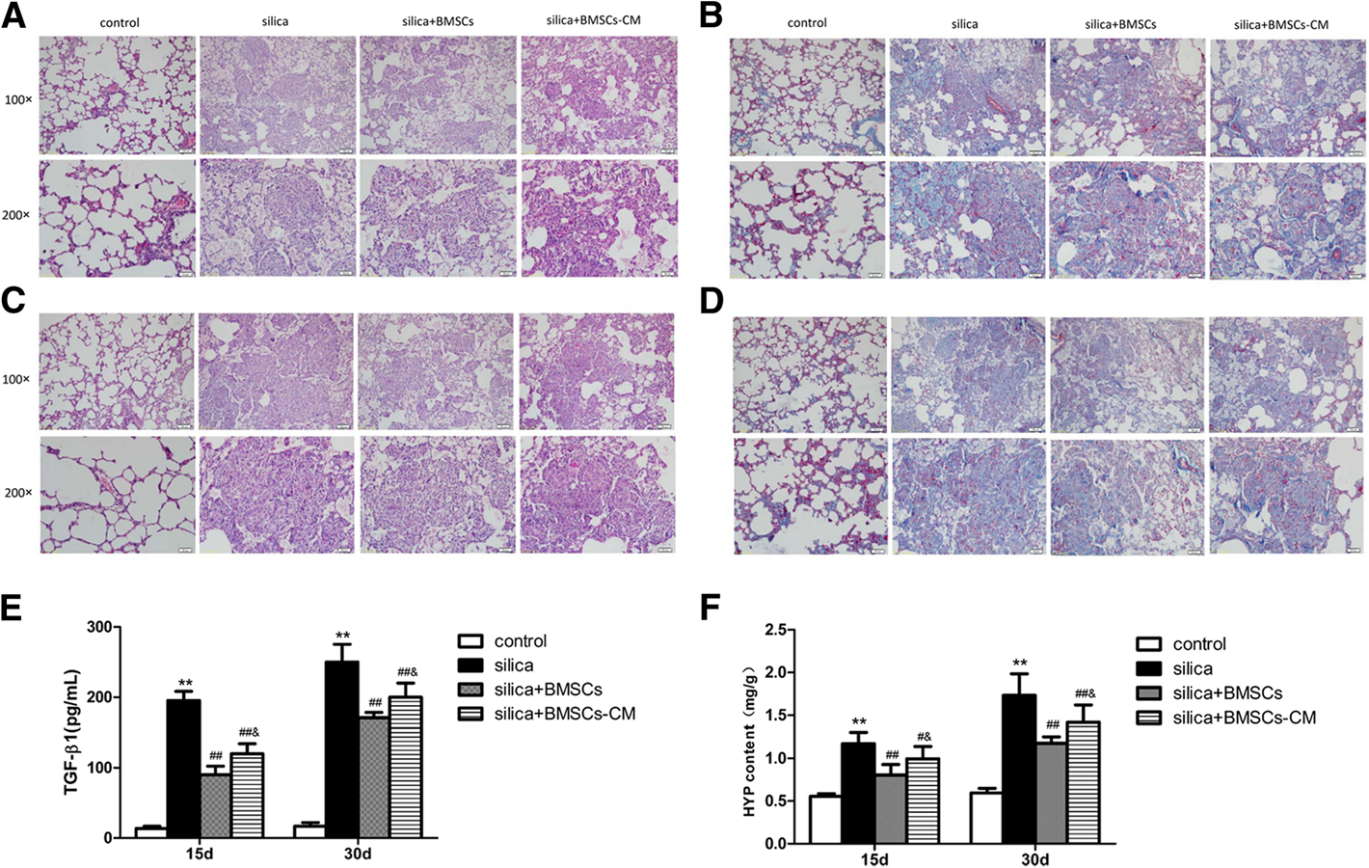
BMSCs-CM inhibited silica-induced pulmonary fibrosis in rats. **a**, **c** H&E staining, (**a**) 15 days after instillation; (**c**) 30 days after instillation. **b**, **d** Masson’s trichrome staining. The result revealed that the collagen deposition (blue areas) was significantly increased in the silica group but decrease in the silica + BMSCs-CM group on 15 (**b**) and 30 days (**d**). Light micrograph magnification × 100 and × 200. Scale bar, 100 μm and 50 μm. **e**, **f** The levels of TGF-β1(**e**) and content of HYP (**f**) were increased in the silica group compared with those in the control group, but they were decreased after the addition of BMSCs-CM (*p* < 0.01). The data were presented as the means ± SD. **p < 0.01 versus the control group; ^##^*p* < 0.01 versus the silica group; ^&^*p* < 0.05 versus the silica + BMSCs group

Because BMSCs hardly differentiated into epithelial cells, paracrine mechanisms of BMSCs may be involved in repair of damaged lung tissue. Notably, paracrine factors were secreted into BMSCs-CM and proven to be beneficial in acute lung injury and pulmonary fibrosis in a previous study [21]. In silica-induced pulmonary fibrosis, the histopathological changes were attenuated by BMSCs-CM. In the silica + BMSCs-CM group, inflammation, cellular nodules, and collagen fibers in the rat lungs were reduced compared with the silica group on day 15 and day 30 (Fig. 5a, c). In addition, Masson staining revealed that collagen deposition (blue areas) was obviously increased in the silica group compared with the control group, and injection of BMSCs-CM reduced the silica-induced blue-stained areas (Fig. 5b, d).

**Fig. 6:**
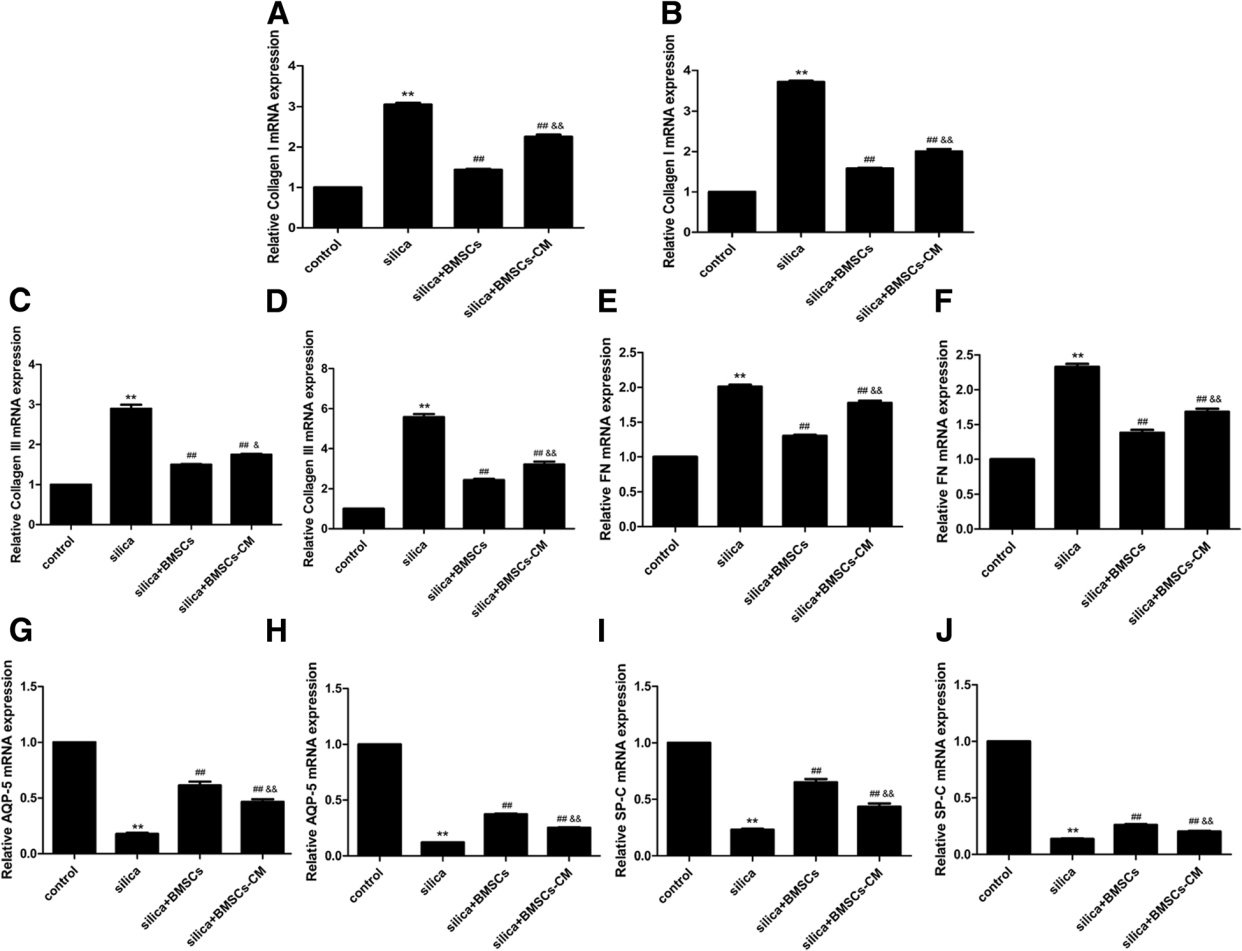
BMSCs-CM decreased the levels of genes for fibrosis and attenuated the injured alveolar epithelial cells. BMSCs-CM attenuate silica-induced pulmonary fibrosis and increase the expression of alveolar epithelial. The fibrosis marker expression of collagen I (**a**, **b**), collagen III (**c**, **d**), and FN (**e**, **f**) in the silica group was significantly increased compared with that in the control group, but was decreased after addition of BMSCs-CM. For the alveolar epithelial markers, either the level of AQP-5 (**g**, **h**) or SP-C (**i**, **j**) was decreased in the silica group compared with that in the control group. But the expression was increased by BMSCs-CM for day 15 (**a**, **c**, **e**, **g**, **i**) and for day 30 (**b**, **d**, **f**, **h**, **j**). The data were presented as the means ± SD. **p < 0.01 versus the control group; ^##^*p* < 0.01 versus the silica group; ^&^*p* < 0.05, ^&&^*p* < 0.01 versus the silica + BMSCs group

TGF-β1 is a pro-fibrotic cytokine in fibrosis diseases, so we evaluated its level in the BALF. We also tested HYP in the lung to observe the content of collagen. The level of TGF-β1 and content of HYP in the silica group were significantly increased than those in the control group (*p* < 0.01). However, after the treatment of BMSCs-CM, they were decreased compared with those in the silica group (*p* < 0.01) (Fig. 5e, f). As expected, qPCR assays showed that silica significantly increased the mRNA expression of collagen I, collagen III, and FN compared with that in the control group, but the expression was decreased in the silica + BMSCs-CM group compared with that in the silica groups (*p* < 0.05; Fig. 6a–f).

BMSCs-CM was also able to improve alveolar epithelial wound repair. AQP-5, which is only expressed on the apical surface of ATI cells, served as a marker for the ATI phenotype. Surfactant protein (SP)-C, produced by ATII cells, was used to identify this cell lineage. The expression of AQP-5 and SP-C in the silica group was significantly decreased compared with that in control group but was increased after addition of BMSCs-CM (*p* < 0.05; Fig. 6g–j).

The above results show that BMSCs-CM protected epithelial cells and inhibited silica-induced pulmonary fibrosis. The protective effect was primarily associated with paracrine functions.

## Discussion

Although studies on BMSCs about migration to target tissues have been reported, there is little data on the distributions of BMSCs in vivo. The present study examined whether systemically applied BMSCs migrated to injured organs, where they moved to, and when they disappeared. For this, NIRF imaging was used to visualize BMSCs migration in living animals. NIRF is an ideal technique that can efficiently penetrate biological tissues with minimal background interference [22, 23]. For NIRF imaging, there are some fluorescent dyes like DiR, a commonly used cyanine dye. BMSCs were labeled with DiR that stains the cytoplasmic membrane of cells [14]. DiR is an effective candidate for NIRF imaging because of its brightness, stability, and lack of effects on cell viability, development, or basic physiological properties. Berninger et al. [24] reported that DiR caused no significant reduction in viability below 10 μg/ml, and we found that 5 μg/ml DiR was the optimal concentration in our study.

The distribution of BMSCs in vivo was sufficiently visualized by NIRF at 750/780 nm. In the control group, many intravenous BMSCs quickly accumulated in the lung at 6 h post-injection. This phenomenon may be explained by the large size of BMSCs (range from 16 to 53 μm; mean 30 μm), which leads to their entrapment in the pulmonary capillary beds [25, 26]. Following intravenous delivery, the lung engraftment efficiency of BMSCs (up to 80%) was very high and few cells reached the systemic circulation [27]. Compared with the control group, many more DiR-labeled BMSCs were preferentially homed to the injured lung tissues and stronger fluorescence intensity was detected at all time points in the silica group. The different accumulation of BMSCs between the two groups was closely related to the presence of an injury process. Consistent with a previous report [8], intravenously infused BMSCs preferred to migrate into silica-injured sites. Thus, the questions arise as to how these BMSCs efficiently home to the target tissues and exert functional effects. The process appears to be associated with multiple biological signals including chemokine receptors, adhesion molecules, proteases, and growth factor receptors [28]. Notably, certain physiological molecules were considered to be critical for the homing ability of BMSCs [29]. Hepatocyte growth factor (HGF) is one of the most efficient chemotactic factors for MSCs migration. Some researchers found that HGF activated BMSCs migration through the PI3K/Akt pathway [30, 31]. Our previous study showed that high levels of HGF were present in culture medium from BMSCs exposed to silica [12]. Thus, we hypothesized that BMSCs migration observed in the present study may be partly mediated by HGF. However, the chemotactic factors involved in BMSCs migration in silicosis remained to be defined and fully elucidated. Nevertheless, the effects, pulmonary entrapment, and homing feature of BMSCs may allow easy access of therapeutic cells to pathology sites to participate in the repair of damaged lung tissues induced by silica.

For therapeutic efficacy of BMSCs, the delivery route, number of cells, and schedule of administration (e.g., single dose versus repeated doses) should be considered. BMSCs were administered by an intravenous injection into the tail to evaluate their anti-fibrotic effect in silica-induced pulmonary fibrosis. Intravenous infusion is one of the principal delivery routes. This route is considered to be minimally invasive, and simple-to-use, and is the most common mode for MSCs delivery in diverse lung disorders [32, 33]. Furthermore, systemic intravenous transplantation may be a suitable administration route in future clinical scenarios. Another important determinant of therapeutic efficacy is BMSCs dosage. In previous studies on rodent models of acute lung injury, the mean dose of MSCs ranged from 20 to 30 × 10^6^cells/kg [2, 32]. In a rat model of silicosis, Zhao and colleagues [34, 35] systemically infused various amount of BMSCs (1~3 × 10^6^cells/rat) to investigate their protective potential. Considering the average weight of a rat, we set the maximum number of administered BMSCs at 4 × 10^6^ BMSCs/rat. In attempts to improve the better beneficial effect, larger numbers of cells were used in some studies. However, higher doses of BMSCs lead to the formation of microemboli-like aggregates [36]. An alternative approach is a second dose during the developmental phase of pulmonary fibrosis. In ventilator-induced lung injury, the dose regimen, intravenous administration of 2 × 10^6^ BMSCs followed by a second dose, was safe and effective for enhancement of lung repair without adverse effects [37]. Meanwhile, the optimal time for repeated cell administration needs to be established. NIRF imaging showed that the DiR fluorescence intensity reached a peak at 6 h after BMSCs transplantation and declined dramatically by day 3. Other researchers found that BMSCs may reach their therapeutic peak and produce soluble factors to ameliorate pulmonary fibrosis during 2~3 days [38]. Based on these findings, a second dose of BMSCs was administered 3 days later (4 days after silica instillation). The results showed that BMSCs (2 × 10^6^ cells, days 1 and 4) clearly reduced the extent of fibrosis in histologic analyses. Furthermore, biomarkers such as collagen I, collagen III, FN, and hydroxyproline were decreased compared with the silica group. These findings indicate that administration of 2 × 10^6^ BMSCs at days 1 and 4 was the optimal dose and timing.

Injury to alveolar epithelial cells was shown to contribute to the pathogenesis of silica-induced pulmonary fibrosis [39]. Any strategy to promote the proliferation or replenishment of damaged alveolar epithelial cells may inhibit pulmonary fibrosis. Some studies have demonstrated that BMSCs adopt the morphological and molecular phenotypes of ATI or ATII cells to repair the damaged lung and reduce pulmonary fibrosis [8, 40]. However, these phenotypes were not observed in our study. As indicated by the in vivo findings, BMSCs homed to the injured lungs, but stayed briefly and disappeared completely after 30 days in silica-instilled rats. Furthermore, none of the BMSCs adopted specific alveolar epithelial phenotypes. These findings were consistent with another study [12]. Our results suggested that differentiation may not be the major mechanism for BMSCs-mediated tissue repair. In fact, the concept for BMSCs engraftment and differentiation is doubtful not only in lung diseases, but also in multiple other diseases. Thus, quite a few studies have indicated that the beneficial effects of BMSCs may be related to paracrine mechanisms. Conditioned media from BMSCs contained various soluble factors that exerted powerful cytoprotective, anti-inflammatory, and anti-fibrotic effects. In our previous study, high concentrations of HGF, keratinocyte growth factor (KGF), and bone morphogenetic protein-7 (BMP-7) were observed in the medium from the silica + BMSCs group [12]. All of the components played crucial roles by accelerating alveolar epithelial cell proliferation and reversing the process of lung fibrosis. In the present study, BMSCs-CM had the ability to protect the alveolar epithelium against silica-induced damage and inhibit the fibrotic process. The histological findings in the lungs, such as cellular nodules, alveolar interstitial thickening, and collagen disposition, tended to be decreased in the silica + BMSCs-CM group compared with the silica group. Correspondingly, the gene expression of collagen I, collagen III, and FN was also downregulated. The expression of alveolar epithelial markers (AQP-5and SP-C) was significantly upregulated in lung tissues treated with BMSCs-CM. Furthermore, BMSCs-CM protected damaged epithelial cells and attenuated silica-induced pulmonary fibrosis. Taken together, the in vivo and in vitro data converge to suggest that the protective effects of BMSCs may not be attributed to differentiation into lung cell phenotypes, but instead rely on paracrine mechanisms through released factors to alleviate the lung injury induced by silica.

## Conclusion

**Fig. 7:**
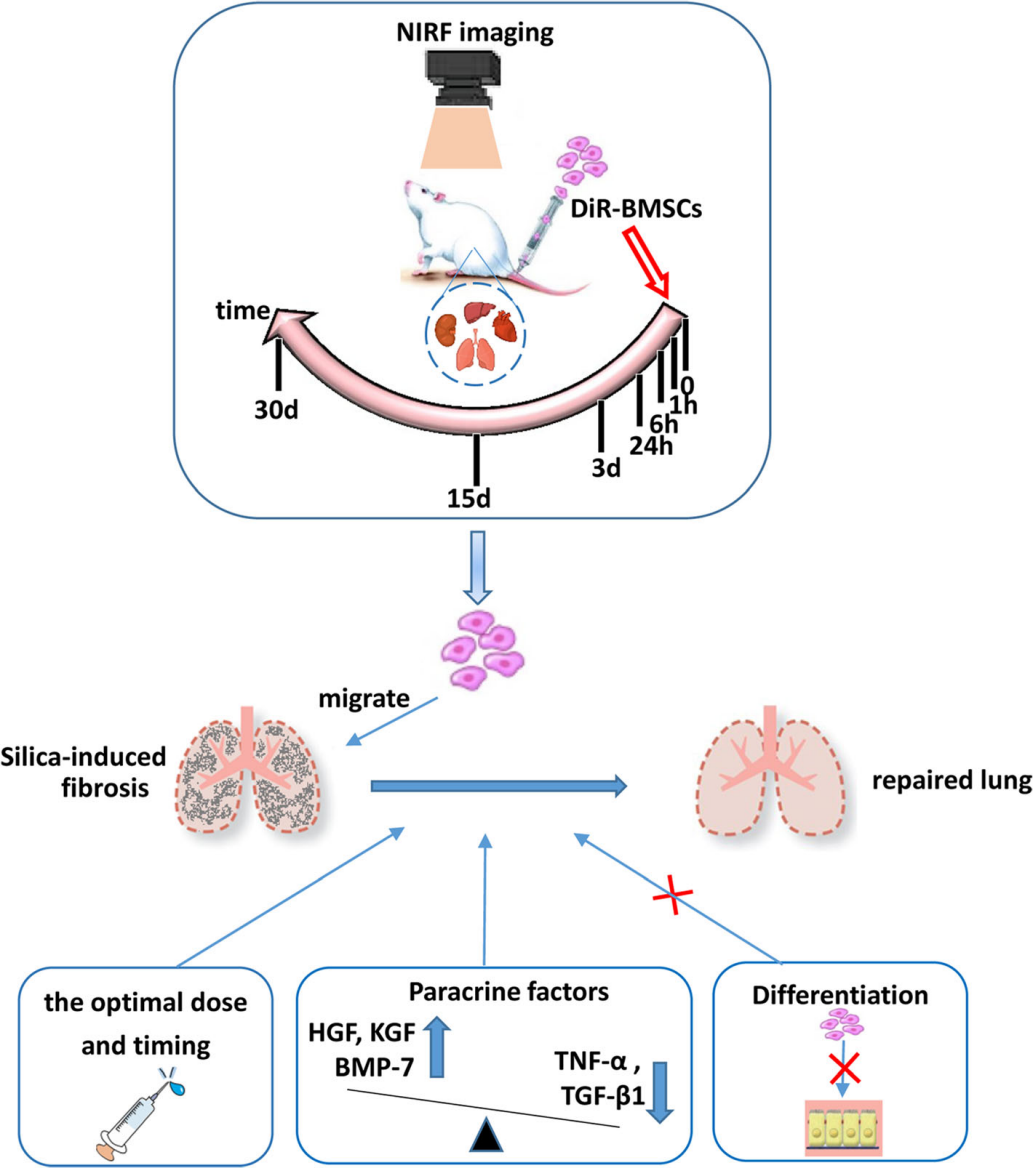
Graphical abstract of our research. BMSCs could migrate to the injured lungs and inhibit silica-induced pulmonary fibrosis. The mechanism may be due to paracrine action rather than the potential of BMSCs to differentiate into alveolar epithelial cells

In summary, our findings show that intravenously delivered BMSCs can migrate to the injured lungs and inhibit silica-induced pulmonary fibrosis (Fig. 7). The most effective administration dose and optimal timing were 2 × 10^6^ BMSCs at days 1 and 4 after silica exposure. Furthermore, the mechanism for amelioration of pulmonary fibrosis may be mediated by paracrine actions rather than by the potential of BMSCs to differentiate and replace the damaged alveolar epithelial cells.

## Additional file

Additional file 1: The findings for isolation and characterization in BMSCs. BMSCs exhibited a homogenous spindle-shaped morphology and expressed markers CD44, CD90, CD 11b, and CD45. Fat droplets and calcium were observed, indicating that cultured BMSCs had a strong ability to differentiate into adipogenic and osteogenic mesenchymal lineages. (DOCX 19 kb)

## Abbreviations

AQP-5: Aquaporin-5
ATI: Alveolar epithelial type I
ATII: Alveolar epithelial type II
BMSCs: Bone marrow mesenchymal stem cells
BMSCs-CM: BMSCs-conditioned medium
BALF: Bronchoalveolar lavage fluid
BMP-7: Bone morphogenetic protein -7
FN: Fibronectin
HGF: Hepatocyte growth factor
HYP: Hydroxyproline
KGF: Keratinocyte growth factor
NIRF: Near-infrared fluorescence
qPCR: Quantitative real-time PCR
SP-C: Surfactant protein-C
Sry: Sex-determining region Y
