# Supplementary material for: Targeted migration of bone marrow mesenchymal stem cells inhibits silica-induced pulmonary fibrosis in rats
Source: Stem Cell Res Ther. 2018 Dec 4;9:335. doi: 10.1186/s13287-018-1083-y (PMC6280342; doi:10.1186/s13287-018-1083-y)
Supplement: Supplementary file 1 — The findings for isolation and characterization in BMSCs. BMSCs exhibited a homogenous spindle-shaped morphology and expressed markers CD44, CD90, CD 11b, and CD45. Fat droplets and calcium were observed, indicating that cultured BMSCs had a strong ability to differentiate into adipogenic and osteogenic mesenchymal lineages. (DOCX 19 kb) [file 13287_2018_1083_MOESM1_ESM.docx]

Additional file 1

**Result**

**Isolation and characterization of BMSCs**

Primary cultured BMSCs exhibited a homogeneous spindle-shaped morphology and expressed markers CD44, CD90, CD 11b, and CD45 at 99.1%, 97.1%, 4.5% and 2.1%, respectively. These findings were consistent with the characteristics of surface antigen of BMSCs. Fat droplets and calcium were observed, indicating that cultured BMSCs had a strong ability to differentiate into adipogenic and osteogenic mesenchymal lineages (data not shown). These findings were described in our previous report [1].

Reference:

1. Li X, An G, Wang Y, et al. Anti-fibrotic effects of bone morphogenetic protein-7-modified bone marrow mesenchymal stem cells on silica-induced pulmonary fibrosis. Exp Mol Pathol. 2017; 102:70-77.
